# Use of a Cationic Emulsion of Latanoprost to Treat Glaucoma Patients with Ocular Surface Disease: A Preclinical Review

**DOI:** 10.1089/jop.2022.0155

**Published:** 2023-05-10

**Authors:** Philippe Daull, Jean-Sébastien Garrigue, Hong Liang, Christophe Baudouin

**Affiliations:** ^1^ Santen SAS, Evry, France.; ^2^ Department of Ophthalmology, Quinze-Vingts Hospital, Paris, France.

**Keywords:** cationic emulsion, latanoprost, ocular surface disease, tear film

## Abstract

Prostaglandin analogue topical medications are one of the most effective therapeutic approaches for the chronic management of glaucoma and ocular hypertension, through the reduction of elevated intra ocular pressure (IOP). While many of the first generations of anti-glaucoma eye drops were preserved with benzalkonium chloride, their repeated use may induce chronic ocular surface toxicity that leads to ocular surface disease (OSD) signs and symptoms. As a result, soft-preservatives and preservative-free formulations have been developed with the goal to avoid the long-term iatrogenic toxicity of the preservative agents. In addition, it has been suggested that OSD and its associated inflammation may negatively impact the efficacy of the IOP-lowering medications, including treatment adherence and compliance. Hence, it may be particularly interesting that glaucoma medications can concomitantly protect and “heal” the ocular surface and its environment while lowering elevated IOP, for the greater benefit of glaucoma patients. The objective of the present review is to briefly present the preclinical data of the cationic oil-in-water emulsion of latanoprost (latanoprost-CE) to shed some light on its mechanisms of action. It overall supports the following hypothesis: the restoration of a healthy ocular surface environment and treatment of the OSD signs and symptoms will allow for an improved elevated IOP reduction and glaucoma management. This would be achieved with a once daily dosing regimen to preserve glaucoma patients' vision, ocular surface, and quality-of-life and wellness.

## Introduction

Glaucoma is a worldwide leading cause of irreversible vision loss.^1^ Glaucoma is an optic neuropathy that leads to irreversible visual field loss and blindness if left untreated. It is estimated that 3.5% of the global population aged 40–80 years have any glaucoma, with the primary open-angle glaucoma (POAG) and the primary angle-closure glaucoma being the most prevalent forms.^2^ Glaucoma affects more than 70 million people worldwide with ∼10% being bilaterally blind, making it the leading cause of irreversible blindness in the world.

The pathogenesis of glaucoma is not fully understood. The level of intraocular pressure (IOP) is related to retinal ganglion cell death; generally, the higher the IOP the more the cell death, and reduction of IOP is the only proven method to treat the disease.

Increased IOP results from a decreased elimination rate of aqueous humor constantly produced by the ciliary body. Hence, either decreasing the production of aqueous humor or increasing the drainage of the aqueous humor will lead to a decreased IOP. The vast majority of glaucoma patients worldwide use anti-glaucoma eye drops formulated with β-adrenergic antagonists, carbonic anhydrase inhibitors, α-adrenergic agonists, prostaglandin analogues (PGA), or a combination thereof.^3–5^

These eye drops are either preserved (with benzalkonium chloride [BAK] or other preservative agents) or preservative-free. PGAs primarily increase the flow of aqueous humor through the uveoscleral pathway and may also act on the trabecular meshwork and relax the ciliary muscle, hence decreasing IOP.^6–9^ PGAs are currently the standard of care and first-line treatment for POAG. They are among the most potent and efficacious medical therapies for lowering elevated IOP.

The poor compliance associated to the use of IOP-lowering eye drops is often due to the ocular complications associated with their chronic use.^10^ The presence of preservative agents, such as BAK, induces iatrogenic toxicity with signs and symptoms of ocular surface disease (OSD) such as dry eye disease (DED).^11^ They lead to decreases in tear film (TF) stability and TF break-up time, Schirmer test values, and worsening of ocular surface inflammation and in DED symptom scores, as evaluated with the OSD index scale, and staining scores.

It is hypothesized that the inflammation starting at the ocular surface and diffusing toward deeper ocular tissues such as the trabecular meshwork following the long-term use of preserved IOP-lowering medications induces a vicious cycle of ocular alterations worsening the glaucoma condition and further jeopardizing the long-term outcome of the disease management.^12,13^ Indeed, there is a long history of evidence that a large population of glaucoma patients also presents OSD signs and symptoms, that is, close to 60% of medically treated glaucoma patients also have DED.^14–16^

As a consequence, preservative-free anti-glaucoma eye drops were developed that appear to be better tolerated than the preserved formulations, and provide a significantly higher patient satisfaction.^17–19^ However, it is noteworthy that although the preservative-free formulations of PGA are considered non-inferior when compared with their preserved reference, none formally demonstrated benefits on OSD and showed numerically lower levels of IOP reduction.^17^

There is also evidence that OSD and ocular surface inflammation negatively affect the efficacy of the anti-glaucoma treatment, and that DED signs management may result in improved IOP control.^20,21^ Removing the preservative agents in preservative-free anti-glaucoma eye drops halts the worsening of the underlying ocular surface inflammation. However, these formulations do not possess “healing” properties *per se*.

It may be reflected by their less than ideal “equivalent” IOP performance and the need for concomitant use of an effective OSD treatment option for the management of OSD signs and symptoms, such as artificial tears. Multiplying number of treatments, that is, adding repeated instillations of artificial tears eye drops on top of the anti-glaucoma eye drops, might also have some deleterious outcomes in term of patient compliance and adherence.

A stable preocular TF is a hallmark of ocular health.^22^ The TF has a vital role in providing lubrication and protection to the ocular surface, as well as maintaining a smooth, refractive surface for optimal visual performance.^23^ A stable state of equilibrium (ie, homeostasis) of the ocular surface with a stable TF and TF lipid layer (TFLL) is expected to create a healthy ocular surface environment that can contribute to the management of the underlying ocular surface inflammation existing in glaucoma patients with OSD and consequently to a better IOP control in these patients.^24^

The Novasorb^®^ technology of cationic oil-in-water emulsions that have demonstrated their excellent performance for the management of mild/moderate and severe DED signs and symptoms, and their capacity to effectively deliver lipophilic drugs to the ocular surface tissues seem to be the ideal drug delivery system (DDS) platform for the lipophilic prodrug latanoprost.^25–27^

The objective of the present paper is to review the preclinical data of a well-tolerated cationic emulsion of latanoprost (latanoprost-CE) to shed some light on its mechanisms of action and the following hypothesis: the concomitant restoration of a healthy ocular surface environment with less conjunctival and trabecular inflammation may allow for a better IOP control and glaucoma management in glaucoma patients with OSD; and this with a convenient once-daily dosing regimen to protect glaucoma patients vision, and their ocular surface.

## Design of a Safe and Effective Cationic Emulsion of Latanoprost at Lowering Elevated IOP

The Novasorb technology is a DDS designed to mimicry the functional composition of a healthy TF with the aim to be the best vehicle for the effective delivery of lipophilic drugs to the anterior segment tissues of the eye (ie, cornea, conjunctiva, the trabecular meshwork, and iris/ciliary body). This DDS is a cationic oil-in-water emulsion that contains non-polar (mineral and/or medium chain triglyceride oils) and polar (cetalkonium chloride, CKC) lipids, non-ionic surfactants (such as poloxamer, tyloxapol, or polysorbate 80), and the osmoprotectant glycerol.^25,26^

**FIG. 1.**
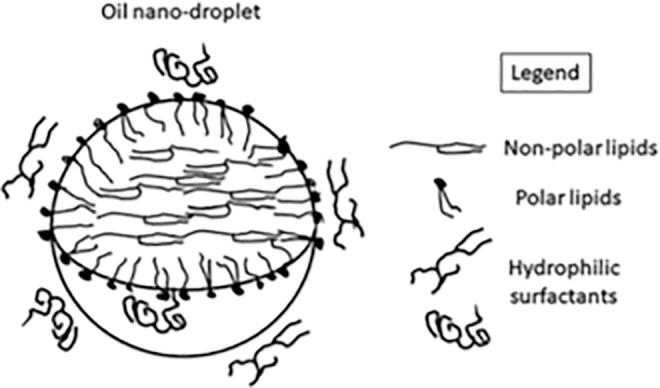
Schematic representation of an oil droplet and its components in the latanoprost-CE vehicle (Adapted from Daull et al., 2020). Latanoprost is a lipophilic molecule with weak surfactant properties, which allow it to localize both within the oil and at the oil/water interface.

The presence of the polar lipid CKC, which is positively charged, brings the cationic feature to the oil nanodroplets of the oil-in-water emulsion. This positive charge contributes to the stabilization of both the drug product (ie, the formulated emulsion) and the TF once it is instilled on the eye. The Fig. 1 illustrates the composition of the cationic oil-in-water emulsion DDS.^28^ Based on the good performances of the cationic oil-in-water emulsions DDS at successfully delivering a lipophilic drug (eg, cyclosporine),^29^ and at restoring a healthy precorneal ocular surface environment (eg, Cationorm^®^),^30,31^ it was decided to use this DDS for the delivery of the lipophilic latanoprost (0.005%) prodrug. The table 1 presents the composition of latanoprost-CE.

**Table 1.** Functional Composition of Latanoprost-Cationic Emulsion

Active ingredient	Percent (w/v)	Function
Latanoprost	0.005	Active ingredient
Excipients		
Oily phase		
Medium chain triglycerides		Oil agent
Cetalkonium chloride		Cationic surfactant
Aqueous phase		
Polysorbate 80		Non-ionic surfactant
Glycerol		Osmotic agent
Water for injection		Solvent

**FIG. 2.**
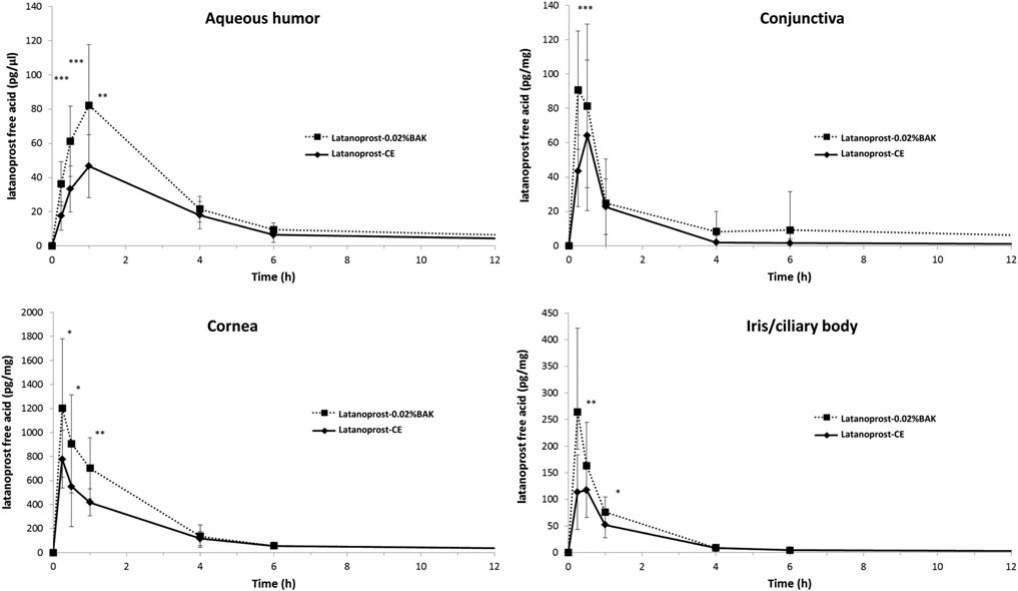
PK profile of latanoprost-free acid after latanoprost-CE and latanoprost-0.02%BAK instillation. PK profile of latanoprost-free acid after a single bilateral instillation of either 0.005% latanoprost-CE or latanoprost-0.02%BAK in the rabbit. Data show the mean concentration – SD. Statistically significant difference between groups: *P < 0.05; **P < 0.001; ***P < 0.0001. There was no statistical difference at 4 and 6 h in the cornea (*P* > 0.53 and *P* > 0.89, respectively) and at 4 and 6 h in the iris/ciliary body (*P* > 0.78 and *P* > 0.99, respectively) between 0.005% latanoprost-CE and latanoprost-0.02%BAK. SD, standard deviation; h, hour; PK, pharmacokinetic. (Adapted from Daull et al., 2012).

The drug delivery efficacy of latanoprost-CE was evaluated through a single dose pharmacokinetic (PK) study in the rabbit and compared with the reference product latanoprost-0.02%BAK (Xalatan^®^).^32^ The ocular tissues distribution was demonstrated to be similar between the 2 latanoprost formulations. A better ocular penetration of the prodrug after latanoprost-0.02%BAK instillation, especially in the cornea and aqueous humor at the shortest time points (up to 1 h post-instillation, Fig. 2), when compared with the concentration levels of latanoprost-free acid reached after the instillation of latanoprost-CE.

This is not surprising, as BAK has been described to be an effective permeation enhancer through its actions on the corneal epithelium. It is noteworthy that there was no statistically significant difference between latanoprost free acid concentrations at the later 4-, 6-, and 24-h post-instillation time points between the 2 latanoprost formulations. It is possible that the lower absorption profile in the rabbit with latanoprost-CE is related to the structure of the rabbit corneal epithelial mucins that are known to be less negatively charged that the ocular surface mucins present on the ocular surface of human beings.^33^

Indeed, the benefits of a prolonged presence of the cationic oil-in-water emulsion in the precorneal space brought by the positive charges at the surface of the oil nanodroplet might be less prominent in species with less negatively charged ocular surface mucins and an ocular surface epithelium with an overall neutral charge. However, it appears that the ocular tissues elimination rate of latanoprost free acid after latanoprost-CE instillation is slower than the one observed after the instillation of latanoprost-0.02%BAK. This is suggestive of a longer residence time of the cationic oil-in-water emulsion, even in the context of a rabbit eye. For both formulations, latanoprost-free acid was barely detectable 24 h post-instillation, and no systemic exposure was detected.

**FIG. 3.**
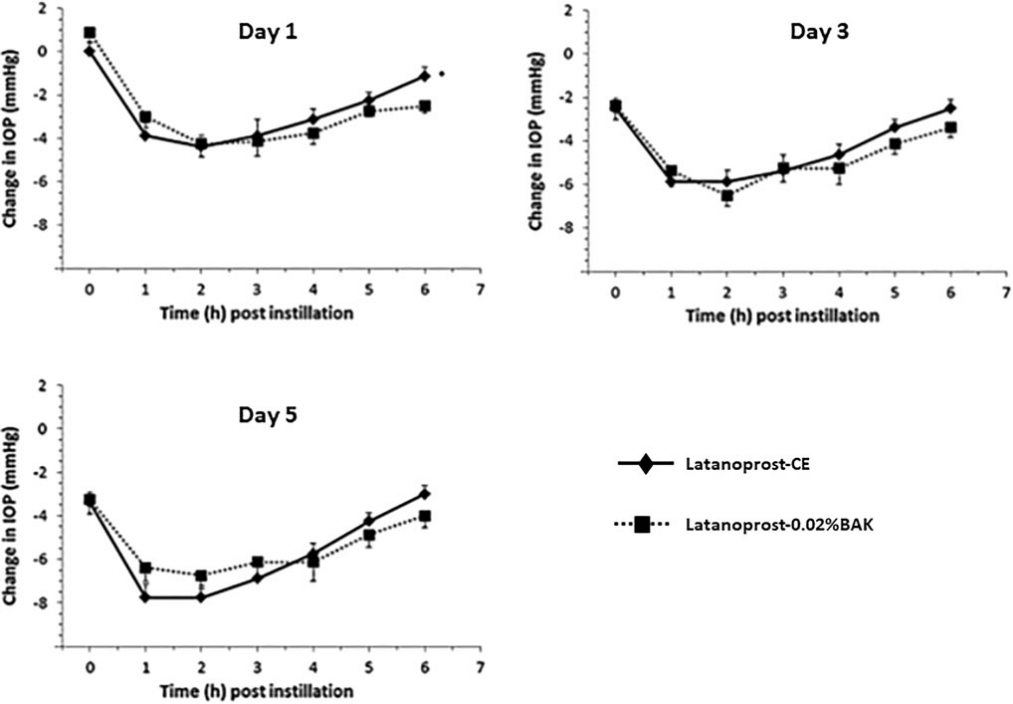
Comparison of the mean change in IOP after daily administrations of latanoprost-CE and latanoprost-0.02%BAK. Comparison of the mean change in IOP in 8 glaucomatous monkey eyes after once-daily administration of latanoprost-CE and latanoprost-0.02%BAK. Data show the mean change in IOP –SEM from vehicle baseline at day 1, 3, and 5. Statistically significant difference in magnitude of IOP reduction between groups: *P < 0.05. IOP on the baseline day and vehicle-treated day was not statistically different (*P* > 0.95) between the latanoprost-CE and latanoprost-0.02%BAK (Adapted from Daull et al., 2012). IOP, intra ocular pressure.

The IOP-lowering efficacy of latanoprost-CE was compared with latanoprost-0.02%BAK in the glaucomatous monkey model.^32^ The formulations were instilled once a day for 5 days and the IOP measured every hour, up to 6 h post-instillation, at days 1, 3, and 5. The comparative IOP changes are presented in Fig. 3. These data show that there was no statistically significant difference between the 2 formulations (except at the 6-h post-instillation time point at day 1), and they demonstrate that both formulations are equally effective at reducing elevated IOP in this preclinical monkey model.

The ocular tolerance of latanoprost-CE was evaluated after twice-daily instillations in the right eye of New Zealand (NZ) white rabbits over a period of 28 days, and it was compared with latanoprost-0.02%BAK.^32^ Table 2 compiles the local tolerance data for both formulations. These data confirmed that latanoprost-CE was well tolerated by a rabbit healthy ocular surface. Although latanoprost-0.02%BAK appears to be also well tolerated in this animal model, it is important to note that there were ∼2 times less conjunctival redness events with latanoprost-CE (57/220) compared with latanoprost-0.02%BAK (98/220).

**Table 2.** Comparison of the Local Tolerance Safety Data for Latanoprost-CE and Latanoprost-0.02%BAK

Treatment daily dose	Control (0.9% NaCl) 2 × 30 μL		Latanoprost-CE 2 × 30 μL		Latanoprost-0.02%BAK 2 × 30 μL	
Number of animals	*M*: 4	*F*: 4	*M*: 4	*F*: 4	*M*: 4	*F*: 4
Body Weight (g^a^)Food consumption (g/day)	2713 ± 106	2755 ± 154	2628 ± 229	2785 ± 117	2740 ± 133	2770 ± 151
Local tolerance (treated eyes)^b,c^						
Conjunctiva redness	132.9 ± 17.4	128.7 ± 15.7	131.0 ± 19.9	142.6 ± 3.8	134.5 ± 6.5	140.6 ± 12.6
Cornea opacity (area)	14/220	20/220	38/220	19/220	32/220	66/220
Iris (reaction)	/	/	/	/	/	/
Ophthalmologic examinations (treated eyes)^a^						
Palpebral and pupillary reflexes	N	N	N	N	N	N
Eye lids irritation and other lesions	A	A	A	A	A	A
Cornea						
Ulcerations	A	A	A	A	A	A
Other lesions	A	A	A	A	+ (1/4)	+ (1/4)
Iris	N	N	N	N	N	N
Lens	N	N	N	N	N	N
Retina	N	N	N	N	N	N
Bio microscopic exam (treated eye)						
Before	N	N	N	N	N	N
Mid treatment	N	N	N	1^*^(1/4)	N	N
End of treatment	1^*^(1/4)	N	N	N	2^*^(1/4)	2^*^(1/4)
Eye histology^a^						
Untreated (left)	N	3^*^(1/4)	3^*^(2/4)	N	3^*^(1/4)	N
Treated (right)	N	N	N	3^*^(1/4)	N	N
Nasal mucosa (irritation score)^a^						
Untreated (left)	/	/	9.77	10.30	9.25	9.77
Treated (right)	/	/	10.25	11.30	9.25	9.00
Irritation Index (untreated – treated)	/	/	None	Minimal	None	None

^a^ Observation at the end of the 28-day treatment period.

^b^ Number observation over the 28-day treatment period.

^c^ All observations were of level 1 in the grading scale.

^*^ 1 = cornea ulceration (trauma); 2 = cornea neovascularization; 3 = conjunctivitis; in brackets, number of animal.

N, normal; A, absent; +, less than a quarter of surface.

This is of particular interest, since it has been largely described in the literature that the use of latanoprost-0.02%BAK is hampered by a relatively high incidence of hyperemia/red eye, a side effect that has a detrimental consequence on patients' treatment compliance and quality-of-life.^34^ The systemic exposure of latanoprost-free acid was negligible in both groups after the repeated instillations. Only one animal (out of 8) in each group had a quantifiable level of latanoprost-free acid above the lowest limit of quantification (30 pg/mL): 32.1 and 33.9 pg/mL for latanoprost-CE and latanoprost-0.02%BAK group, respectively.

In conclusion, these safety data demonstrate that both latanoprost formulations were overall well tolerated by the healthy ocular surface of NZ white rabbit, with latanoprost-CE inducing less conjunctiva irritation than latanoprost-0.02%BAK. However, since glaucoma patients also present an ocular surface with a damaged corneal epithelium, with 45%–60% of patients with glaucoma using IOP-lowering drugs have some degree of OSD,^35^ it will be of particular interest to evaluate the impact (ie, the safety) of latanoprost-CE and latanoprost-0.02%BAK on diseased cornea presenting epithelial defects.

It is expected that only a safe formulation will be able to contribute positively to the restoration of a diseased corneal epithelium (See section Efficacy of the cationic emulsion of latanoprost at restoring the corneal epithelium and at managing ocular surface inflammation).

## Efficacy of the Cationic Emulsion of Latanoprost at Restoring a Healthy TF, TFLL and Ocular Surface Environment

**FIG. 4.**
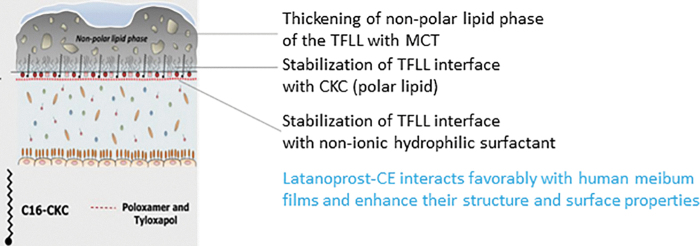
Interaction of the cationic oil-in-water emulsion components with the TF and TFLL. CKC, cetalkonium chloride; MCT, medium chain triglycerides; TFLL, tear film lipid layer; TF, tear film.

Previous data obtained for Cationorm, an “empty” cationic oil-in-water emulsion without any loaded drug, have demonstrated that the cationic oil-in-water emulsion interacts favorably with the TF and TFLL. The instillation of one eye drop of the cationic oil-in-water emulsion was able to restore a diseased TF, by increasing the thickness of the TFLL, stabilizing the interface between the TFLL and the underlying aqueous phase of the TF, as a result of the presence of the polar lipid CKC and non-ionic surfactants mimicking the action of the surface active proteins naturally present in the aqueous phase of the TF.^30,36^ The following Fig. 4 illustrates how the balanced mix of the different components (non-polar and polar lipids, non-ionic surfactant(s), osmoprotectant and water) of the cationic oil-in-water emulsion stabilize the TF.

The non-polar lipids integrate themselves in the non-polar layer of the TFLL, increasing the thickness of the TFLL, whereas CKC, like any polar lipid, localizes at the interface between the oil and water layers of the TF. In addition, in case of polar lipid deficiency, as seen in altered ocular surface TF in patients with OSD, the presence of CKC at the oil/water interface is able to reduce the destabilizing interactions between the non-polar lipids and water molecules and thus prevents TFLL rupture.^37^

Hence, the supplementation with CKC is able to compensate for polar lipid deficiency, and it contributes to the stabilization of the TF. As a consequence, instillations of cationic oil-in-water emulsion eyedrops decrease the surface tension of the TF, which contributes to the stabilization of the TF. A more detailed explanation on how the cationic oil-in-water emulsions contribute to the stabilization of the TF and TFLL has been reviewed elsewhere.^28^

Similar TF stabilization properties were observed with latanoprost-CE, which on instillation, enhanced the structure and surface properties of Meibum gland secretion films.^38^

## Efficacy of the Cationic Emulsion of Latanoprost at Restoring the Corneal Epithelium and at Managing Ocular Surface Inflammation

In an *in vitro* corneal wound-healing model, latanoprost-CE was as effective at supporting wound closure pace as other BAK-free PGAs. Conversely, latanoprost-0.02%BAK appeared to be detrimental and prevented the “re-epithelization” of the scrapped area in this *in vitro* model.^39^ Ki-67 (a marker of proliferative cells) immunohistology staining demonstrated that a higher number of proliferating cells were present in the scrapped area on treatment with latanoprost-CE, when compared with latanoprost-0.02%BAK, or other PGAs with different BAK concentrations. These data by Liang et al. (2022) confirmed that BAK was, in a dose-dependent manner, deleterious for the wound-healing process.

They also demonstrate that the latanoprost-CE can maintain a normal healing process comparable to the one observed on treatment with control PBS. To further analyze the impact of latanoprost-CE instillations on a healthy ocular surface, an established severe rabbit toxicological model (15 instillations over 75 min, one instillation every 5 min) was used. This experiment confirmed that repeated instillations of latanoprost-CE were well tolerated with no signs of ocular surface irritation or lesions as it was observed on treatment with latanoprost-0.02%BAK.

Indeed, the repeated use of BAK-preserved anti-glaucoma medications have demonstrated their long-term toxic and immunoinflammatory effects in the ocular surface's conjunctiva and the inner structure of the trabecular meshwork.^40^ In this severe toxicity model, latanoprost-0.02%BAK was not tolerated by the healthy rabbit ocular surface.

**FIG. 5.**
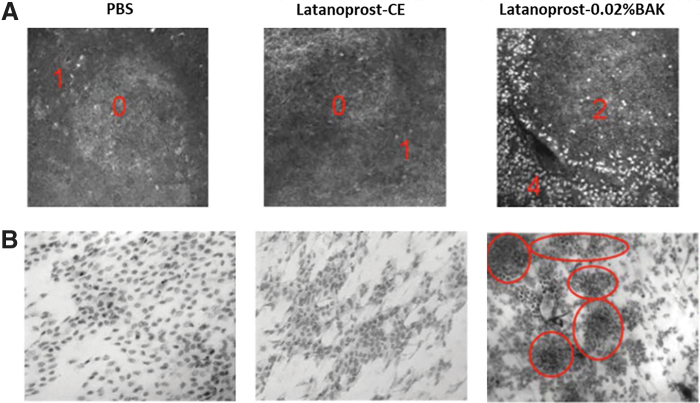
Inflammatory cells in the CALT after treatment with latanoprost-CE and latanoprost-0.02%BAK **(A)** IVCM images of rabbit CALT 4 h after instillations with latanoprost-CE and latanoprost-0.02%BAK eye drops. Numerous inflammatory cells (*white dots*) were observed infiltrating the periphery and center of the CALT structure, especially after the instillation of latanoprost-0.02%BAK. Latanoprost-CE did not induce any obvious inflammatory cell infiltration, as in PBS-instilled eyes. The red numbers indicate the intensity of the infiltration. Bar scale 100 μm. **(B)** Conjunctival impression cytology staining with cresyl violet revealed that PBS and latanoprost-CE eyes presented almost normal epithelial patterns without any obvious inflammatory cell infiltration. By contrast, latanoprost-0.02%BAK induced numerous polymorphonuclear inflammatory cells as islets (*red circles*). (Original size 40 × ). CALT, conjunctiva associated lymphoid tissue. (Adapted from Liang et al., 2009).

Indeed, no infiltrating inflammatory cells (hyper-reflective patterns corresponding to polymorphonuclear leukocytes, lymphocytes and dendriform cells by *in vivo* confocal microscopy, or CD45-positive cells by impression cytology) in the conjunctiva-associated lymphoid tissue (CALT) were observed on latanoprost-CE treatment. In contrast, latanoprost-0.02%BAK treatment resulted in a drastic increase of inflammatory cells count in the CALT, as seen in Fig. 5.^41^

Previous preclinical as well as clinical data indicate that the cationic oil-in-water emulsion (Cationorm and Cationorm Pro; Santen Pharmaceuticals), and cyclosporine-loaded oil-in-water emulsion (Ikervis^®^ and Verkazia^®^; Santen Pharmaceuticals) were able to promote corneal wound healing in DED patients with ocular signs of OSD and vernal keratoconjunctivitis patients.

It was, therefore, interesting to confirm that the good performance observed with the other cationic oil-in-water emulsions on the corneal healing process was also a feature of latanoprost-CE, considering that close to 60% of glaucoma patients have OSD with a damaged corneal epithelium, and that a significant proportion of glaucoma patients treated with BAK-preserved PGAs (such as latanoprost-0.02%BAK) suffer from OSD.^14,42^

In this case, pre-existing OSD conditions are generally worsened and exacerbated by the repeated use of the BAK-preserved antiglaucoma drugs. The ocular surface of these patients generally displayed subclinical to clinical signs of inflammation leading to the loss of goblet cells and to a dramatic reduction of the production of protective mucins (eg, secreted mucin MUC5AC).^43^ Moreover, as the treatment proceeds, the corneal surface is wounded by the repeated applications of eye drops containing 0.02% BAK.

Apoptosis of corneal epithelial cells develops, leading to the formation of scars accompanied by irritation and itching sensations that led the patients to end their treatment and have a poor quality-of-life and treatment experience, and are at risk of becoming blind.

In an *in vitro*, as well as in a rat model of corneal debridement, it appeared that latanoprost-CE was able to improve the wound-healing pace, when compared with the control.^44^ Treatments with latanoprost-CE or its vehicle (CE) allowed for a complete and almost scar-free re-epithelization of the cornea. It is of particular notice that the remaining scar after treatment with latanoprost-CE was approximately one-third of the one observed after PBS treatment.

**FIG. 6.**
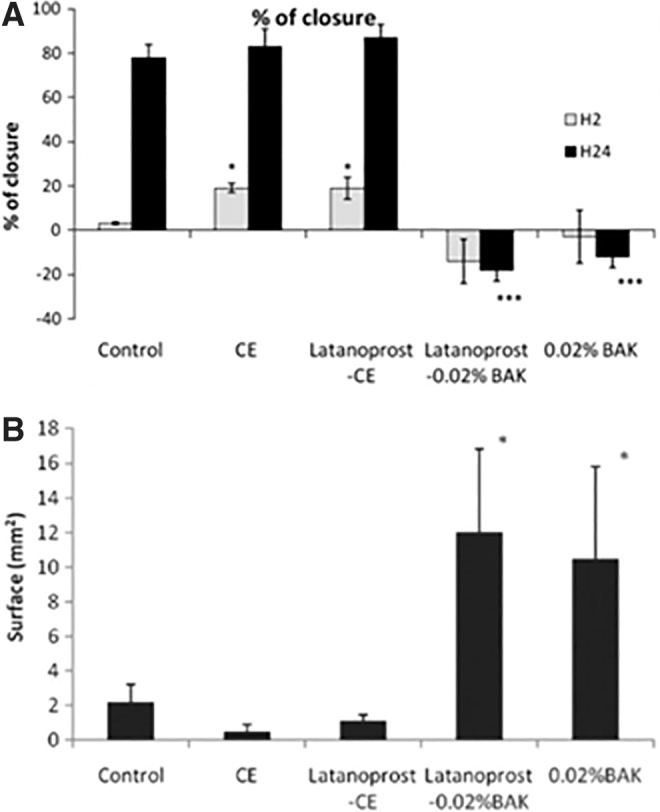
Effects of latanoprost-CE and latanoprost-0.02%BAK on the healing process in vitro and in vivo. **(A)** In vitro: Percentage of wound closure at H24 (expressed as a percentage of H0 scrape distance) after the different treatments of a scrapped HCE cell layer. *P, 0.03 compared with control, ***P, 0.0001 compared with control. **(B)** In vivo: Plotted rat cornea scar (opaque area) surface measured *in vivo* by slit-lamp biomicroscopy evaluation at day 5 after the different treatments The opaque lesion surface of the rat cornea shows that the largest area was observed for latanoprost-0.02%BAK and the 0.02% BAK solution. Latanoprost-CE and its CE vehicle present the smallest opaque area, even when compared with PBS. *P, 0.05 compared with control. (Adapted from Liang et al., 2012). HCE, human corneal epithelium.

**FIG. 7.**
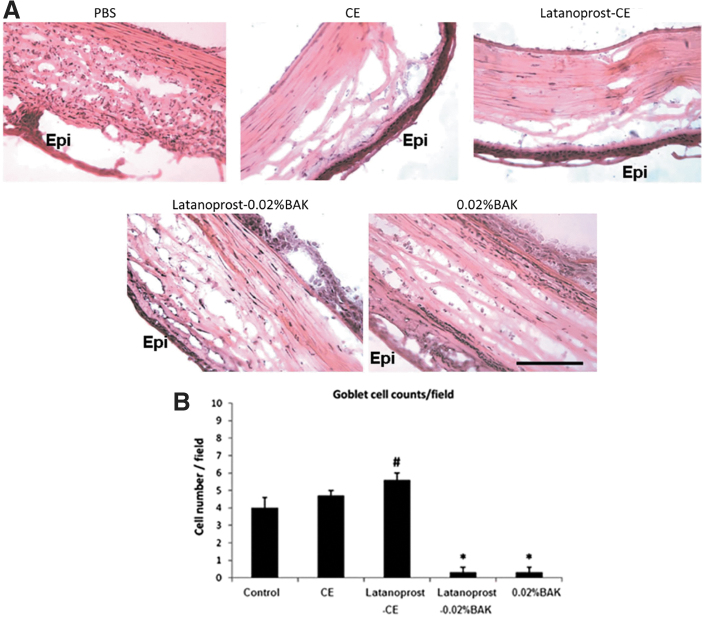
**(A)**. Effects of latanoprost-CE on inflammatory cells and MUC5AC conjunctival expression **(A)** Hematoxylin–eosin staining of rat corneas after the different treatments. Numerous infiltrated inflammatory cells can be observed with both latanoprost-0.02%BAK and the 0.02%BAK solution. “Epi” localizes the epithelium (scale bar = 100 μm). **(B)** Effects of latanoprost-CE on inflammatory cells and MUC5AC conjunctival expression **(B)** Goblet cell counts per field. Note the disappearance of goblet cells after treatment with the 2 BAK-containing solutions. *P, 0.0001 compared with control. ^#^*P*, 0.02 compared with control. (Adapted from Liang et al., 2012).

This suggests that latanoprost-CE was able to promote a safe healing process of the diseased cornea. By contrast, latanoprost-0.02%BAK aggravated the size of the lesion *in vitro*, and resulted in the formation of scar tissue within the reconstructed rat corneal epithelium (Fig. 6).^44^ This *in vivo* experiment in the rat also demonstrated that a lower number of inflammatory cells infiltrated the debrided cornea epithelium after latanoprost-CE treatment, and that both the cationic emulsion vehicle and latanoprost-CE treatments were able to maintain conjunctiva goblet cells' expression of mucin MUC5AC (Fig. 7).

Interestingly, it appears that the PGA latanoprost has some beneficial effects on mucin MUC5AC's expression by itself, as the MUC5AC immunostaining after latanoprost-CE treatment tends to be stronger when compared with the latanoprost-CE vehicle-treated group^44,45^ An *in vivo* cytoprotective effect of latanoprost seemed also to be observed when the number of goblet cells was analyzed (Fig. 7B), which parallels the *in vitro* cytoprotective effect previously determined on conjunctiva-derived epithelial cells.^46^

These beneficial effects of latanoprost on goblet cells and MUC5AC were completely undetected and annihilated by the toxic effects of BAK when formulated as a preserved eye drop. Mucins are a family of high molecular weight, heavily glycosylated proteins (glycoconjugates) produced by many epithelial tissues. They bind to pathogens as part of the immune system and can retain water and help maintain a wet environment that protects the ocular surface. Collectively, these data demonstrate that Latanoprost-CE possesses potential beneficial properties, that is, it improves corneal wound healing and protects conjunctival goblet cells. On the contrary, latanoprost-0.02% BAK treatment on de-epithelialized cornea worsens the condition of the ocular surface.

These benefits on the corneal wound-healing process and the protection of the goblet cells by latanoprost-CE may find their explanation in the fact that the cationic oil-in-water emulsion are able to protect the ocular surface by: (1) mechanically creating a healthy environment for the ocular surface via the stabilization of the TF and TFLL, and (2) by controlling, via the ancillary pharmacological action of CKC, a known protein kinase Cα (PKCα) inhibitor, the level of ocular surface inflammation.^47^

Indeed, Chen et al. (2010) have demonstrated with a PKCα-knockout mouse exposed to a dry environment, to mimic DED with its corneal epithelium lesions, that blocking PKCα resulted in a lower number of inflammatory cells on the ocular surface and a better wound-healing process of corneal epithelium lesions.^48^

## Efficacy of the Cationic Emulsion of Latanoprost at Improving Signs of OSD

The efficacy at reducing signs of corneal damage [ie, corneal fluorescein staining (CFS) score] of the cationic oil-in-water emulsion loaded with various PGAs was evaluated in a well-described mouse model of dry eye.^49,50^ Mice with a scopolamine transdermal patch placed in a controlled environment chamber were treated for 7 days with various PGA eye drops once dry eye signs peaked at day 3.

**FIG. 8.**
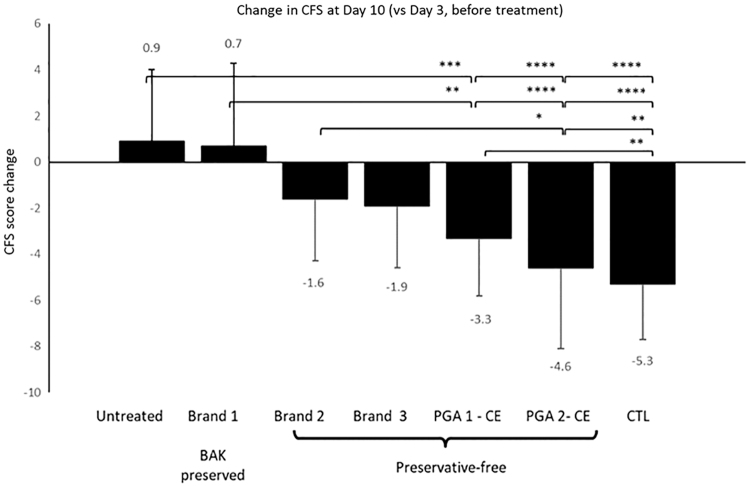
Efficacy of prostaglandin analogues at reducing signs of ocular surface disease in a mouse model of dry eye. Mean reduction in CFS score at day 10 (vs. day 3, before treatment) after treatment with various formulations of PGAs IOP-lowering drugs in the controlled environment mouse model of dry eye. Statistical significance was set at a *P < 0.05, **P < 0.01, ***P < 0.001, ****P < 0.0001. Results are presented as mean ± SD. CE, cationic emulsion; CTL, control; PGA, prostaglandin analogue. (Adapted from Daull et al., 2017).

The mean CFS change at day 10 (vs. day 3, dry eye baseline) demonstrated that the PGAs formulated with the cationic oil-in-water emulsion performed better at reducing CFS scores than BAK-preserved and preservative-free PGA formulations (Fig. 8). The cationic oil-in-water emulsion of PGAs was almost as effective as the positive control formulation aimed at healing the ocular surface.^27^ These data are corroborated by previous similar studies using cationic oil-in-water emulsion vehicles loaded with cyclosporine and developed for the management of severe DED signs.^51^

## Translation of Preclinical *In Vitro* and *In Vivo* Efficacy Evidence to Glaucoma Patients

The preclinical study results described so far demonstrate that latanoprost-CE possesses interesting features that define it as an effective drug formulation for reducing elevated IOP in glaucoma patients suffering from OSD: (1) latanoprost-CE is able to delivers an effective dose of latanoprost-free acid to the target tissue, (2) latanoprost-CE is as effective as the PGA gold standard antiglaucoma eye drop at reducing elevated IOP, (3) latanoprost-CE appears to have an improved local tolerance profile compared with latanoprost-0.02%BAK, (4) latanoprost-CE is able to mechanically improve the TF and TFLL stability and film properties and restore a healthy ocular surface environment, (5) latanoprost-CE is able to contribute effectively in the management of ocular surface inflammation, (6) latanoprost-CE improves the healing process of damaged cornea, and (7) latanoprost-CE is able to improve CFS scores in the context of dry eye.

However, the remaining question is the following: do all the aforementioned properties translate to the clinic? And is latanoprost-CE able to treat concomitantly elevated IOP and OSD signs and symptoms in glaucoma patients while preserving their compliance and well-being and improving their treatment satisfaction and experience?

In a phase II clinical study comparing latanoprost-CE with the soft-preserved Travatan^®^Z (0.004% travoprost ophthalmic solution, Alcon Laboratories), it appeared that IOP change from baseline for latanoprost-CE was non-inferior to TravatanZ, with a trend toward a superior IOP reduction at each time point at month 3: −7.2 ± 2.9 versus −6.0 ± 3.0, −6.7 ± 3.0 versus −5.9 ± 3.5, −6.0 ± 3.3 versus −5.4 ± 3.7 at 8:00 AM, 10:00 AM, 4:00 PM for latanoprost-CE and TravatanZ, respectively.^27^

This trend for a better IOP management is not unexpected, considering that it has been described that reducing ocular surface inflammation in glaucoma patients is accompanied by better IOP outcomes.^20,21^ A similar trend toward better CFS scores (modified Oxford scale) and TF break-up times with latanoprost-CE was also observed when compared with TravatanZ (BAK-free, soft-preserved with an ionic buffered system).

However, these promising results will need to be confirmed in a larger clinical trial. The results of the comparison of latanoprost-CE to latanoprost-0.02%BAK (Xalatan, Pfizer) in a phase III clinical trial on both IOP management and on OSD signs and symptoms tend to support our hypothesis (data on file).

## Discussion & Conclusion

OSD is a very common comorbidity associated to glaucoma, as close to 60% of glaucoma patients suffer from OSD signs and symptoms of various severity.^14,42^ The negative impact preservative agents have on the ocular surface, extensively described for BAK, due to its long history of use to preserve eye drops, is largely accepted worldwide, and the recent development of preservative-free eye drop formulations is just the clear confirmation that such molecules should be avoided.^52^

However, this should not prevent the ophthalmic community from maintaining its watch on the excipients that made their apparition in these preservative-free formulations, as their ocular impact after long-term use is not yet well established and since some recent *in vitro* studies suggest pro-inflammatory response and cytotoxicity in corneal cells with these excipients (eg, macrogolglycerol hydroxystearate 40).^53^

Preservative-free anti-glaucoma eye drops have greatly benefited glaucoma patients, and not only those presenting signs and symptoms of OSD. They contributed to their elevated IOP management, while reducing the discomfort associated to the repeated instillations. However, for a better long-term vision protection outcome, it is important that the IOP reduction is as important as possible, and unfortunately, most of the preservative-free “generic-like” PGA formulations consistently show a lower IOP reduction performance, while remaining within the 1.5 mmHg margin at each time point.

This might be explained by the fact that preservative agents, such as BAK, are also effective permeation enhancers. This is nicely demonstrated by the backward reformulation of 0.03% bimatoprost/0.005% BAK to 0.01% bimatoprost/0.02% BAK while the IOP lowering efficacy was maintained within the non-inferiority margin. It is also possible that while being preservative-free, these PGA formulations are unable to address the underlying inflammation of the ocular surface that is known to negatively impact elevated IOP management outcomes; in the context of anti-glaucoma eye drop treatment, with possible trabecular meshwork inflammation and damage, or filtering surgery.

Removing preservatives such as BAK from PGA formulations may not be enough to improve OSD and may still require the concomitant use of artificial tears to effectively alleviate both signs and symptoms. This polymedication, while certainly effective, requires the repeated instillation of various eye drops during the day, which can negatively impact glaucoma patients' quality-of-life and treatment experience, if not their compliance.

The latanoprost-CE described in these pages takes advantage of the Novasorb technology of the cationic oil-in-water emulsion, an eye drop formulation that mimics a healthy TF that helps mechanically restore a safe TF and ocular surface environment and which is at the same time an effective DDS for the delivery of the lipophilic prodrug latanoprost.^27,28^

The latanoprost-CE was demonstrated to protect the ocular surface through: (1) the restoration of a thick and stable TFLL via oil supplementation, (2) the stabilization of the oil/water interface between the TFLL and the aqueous phase of the TF by bringing optimized amounts of the cationic polar lipid agent (ie, CKC) and a non-ionic surfactant (polysorbate 80). The resulting restored TFLL protects the TF from evaporation, thus restoring a healthy ocular surface environment that contributes to the homeostasis of the ocular surface.^30,54^

And in addition to these mechanical beneficial effects on the TFLL and TF stabilization, the cationic oil-in-water emulsion is also able to promote the healing process of the corneal epithelium via, up to a certain extent, the management of the ocular surface inflammation (ie, by controlling the inflammatory cell recruitment and modulating pro-inflammatory mediators).^47^

This wealth of preclinical evidence explains the excellent clinical outcomes observed in the phase II clinical trial, and it corroborates the hypothesis postulated by Batra et al., (2014) and Dubrulle et al., (2018). The improvement of the ocular surface environment and its inflammation concomitant with an IOP-lowering drug translate into a better IOP lowering efficacy, possibly through decreased inflammation of both conjunctiva and trabecular meshwork among other eye tissues leading to improved outflow facility of the eye.

Hence, the simultaneous treatment of the ocular surface while treating elevated IOP with the same eye drop formulation may represent the next generation of glaucoma eye drop treatment with possibly a better quality-of-life and treatment experience for glaucoma patients.^20,27^ Based on cost-utility analyses over a 5-year time horizon, the latanoprost-CE resulted in an incremental 0.35 Quality-Adjusted Life Years compared with Latanoprost.^55^

Similarly, it was concluded as potentially highly cost-effective versus standard latanoprost formulation for glaucoma-OSD patients from the Italian National Health Service perspective.^56^ These results should be confirmed by future economic evaluations carried out alongside empirical trials.
